# Do Infectious Diseases After Kidney Retransplantation Differ From Those After First Kidney Transplantation?

**DOI:** 10.1093/ofid/ofae055

**Published:** 2024-02-06

**Authors:** Katharina Kusejko, Dionysios Neofytos, Christian van Delden, Hans H Hirsch, Pascal Meylan, Katia Boggian, Cedric Hirzel, Christian Garzoni, Daniel Sidler, Aurelia Schnyder, Stefan Schaub, Déla Golshayan, Fadi Haidar, Marco Bonani, Roger D Kouyos, Nicolas J Mueller, Peter W Schreiber, the Swiss Transplant Cohort Study, P Amico, J-D Aubert, V Banz, S Beckmann, G Beldi, C Berger, E Berishvili, A Berzigotti, I Binet, P-Y Bochud, S Branca, H Bucher, E Catana, A Cairoli, Y Chalandon, S De Geest, O De Rougemont, S De Seigneux, M Dickenmann, J L Dreifuss, M Duchosal, T Fehr, S Ferrari-Lacraz, C Garzoni, D Golshayan, N Goossens, F H J Halter, D Heim, C Hess, S Hillinger, H H Hirsch, P Hirt, G Hofbauer, U Huynh-Do, F Immer, M Koller, M Laager, B Laesser, F Lamoth, R Lehmann, A Leichtle, O Manuel, H P Marti, M Martinelli, V McLin, K Mellac, A Merçay, K Mettler, A Müller, N J Mueller, U Müller-Arndt, B Müllhaupt, M Nägeli, G Oldani, M Pascual, J Passweg, R Pazeller, K Posfay-Barbe, J Rick, A Rosselet, S Rossi, S Rothlin, F Ruschitzka, T Schachtner, U Schanz, S Schaub, A Scherrer, A Schnyder, M Schuurmans, S Schwab, T Sengstag, F Simonetta, S Stampf, J Steiger, G Stirnimann, U Stürzinger, C Van Delden, J-P Venetz, J Villard, J Vionnet, M Wick, M Wilhelm, P Yerly

**Affiliations:** Department of Infectious Diseases and Hospital Epidemiology, University Hospital Zurich, Zurich, Switzerland; Institute of Medical Virology, University of Zurich, Zurich, Switzerland; Division of Infectious Diseases, University Hospital of Geneva, Geneva, Switzerland; Division of Infectious Diseases, University Hospital of Geneva, Geneva, Switzerland; Transplantation and Clinical Virology, Department of Biomedicine, University of Basel, Basel, Switzerland; Clinical Virology, Laboratory Medicine/Infectious Diseases, and Hospital Epidemiology, University Hospital Basel, Basel, Switzerland; Faculty of Biology and Medicine, University of Lausanne, Lausanne, Switzerland; Division of Infectious Diseases, Infection Prevention and Travel Medicine, Cantonal Hospital of St Gallen, St Gallen, Switzerland; Department of Infectious Diseases, Inselspital, Bern University Hospital, University of Bern, Bern, Switzerland; Department of Infectious Diseases, Inselspital, Bern University Hospital, University of Bern, Bern, Switzerland; Department of Internal Medicine, Clinica Luganese Moncucco, Lugano, Switzerland; Division of Nephrology and Hypertension, Inselspital, Bern University Hospital, Bern, Switzerland; Clinic for Nephrology, Cantonal Hospital of St Gallen, St Gallen, Switzerland; Clinic for Transplantation Immunology and Nephrology, University Hospital Basel, Basel, Switzerland; Transplantation Center, Lausanne University Hospital, Lausanne, Switzerland; Division of Nephrology, Department of Medicine, University Hospital of Geneva, Geneva, Switzerland; Division of Nephrology, University Hospital Zurich, Zurich, Switzerland; Department of Infectious Diseases and Hospital Epidemiology, University Hospital Zurich, Zurich, Switzerland; Institute of Medical Virology, University of Zurich, Zurich, Switzerland; Department of Infectious Diseases and Hospital Epidemiology, University Hospital Zurich, Zurich, Switzerland; Department of Infectious Diseases and Hospital Epidemiology, University Hospital Zurich, Zurich, Switzerland

**Keywords:** infections, kidney retransplantation, organ allocation

## Abstract

**Background:**

Infectious diseases (IDs) are highly relevant after solid organ transplantation in terms of morbidity and mortality, being among the most common causes of death. Patients undergoing kidney retransplantation (re-K-Tx) have been already receiving immunosuppressive therapy over a prolonged period, potentially facilitating subsequent infections. Comparing ID events after re-K-Tx and first kidney transplantation (f-K-Tx) can delineate patterns and risks of ID events associated with prolonged immunosuppression.

**Methods:**

We included adult patients with records on f-K-Tx and re-K-Tx in the Swiss Transplant Cohort Study. We analyzed ID events after f-K-Tx and re-K-Tx within the same patients and compared infection rates, causative pathogens, and infection sites. Recurrent time-to-event analyses were performed for comparison of infection rates.

**Results:**

A total of 59 patients with a median age of 47 years (range, 18–73) were included. Overall, 312 ID events in 52 patients occurred. In multivariable recurrent event modeling, the rate of ID events was significantly lower after re-K-Tx (hazard ratio, 0.70; *P* = .02). More bacterial (68.9% vs 60.4%) and fungal (4.0% vs 1.1%) infections were observed after f-K-Tx but fewer viral infections (27.0% vs 38.5%) as compared with re-K-Tx (*P* = .11). After f-K-Tx, urinary and gastrointestinal tract infections were more frequent; after re-K-Tx, respiratory tract and surgical site infections were more frequent (*P* < .001).

**Conclusions:**

ID events were less frequent after re-K-Tx. Affected sites differed significantly after f-K-Tx vs re-K-Tx.

## Graphical Abstract

**Graphical abstract:**
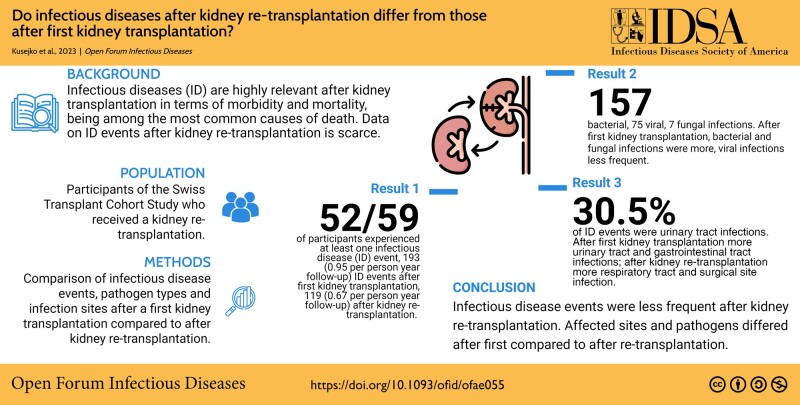
This graphical abstract is also available at Tidbit: https://tidbitapp.io/tidbits/do-infectious-diseases-after-kidney-re-transplantation-differ-from-those-after-first-kidney-transplantation/update

After failure of an initial kidney graft, kidney retransplantation (re-K-Tx) can be a strategy to avoid permanent renal replacement therapy [1]. Studies on re-K-Tx predominantly focused on graft and recipient survival [1–3]. The majority of studies investigated differences in transplant-related outcomes by comparing a population of patients after first kidney transplantation (f-K-Tx) with a population after re-K-Tx. Data on infectious disease (ID) events after re-K-Tx are generally scarce. Most studies in this field focused on retransplantation due to BK virus nephropathy [4–9].

Infections remain one of the most frequent causes of death among transplant recipients [10–12]. A recent monocentric German study indicated poorer graft and patient survival after re-K-Tx as compared with a matched control population after f-K-Tx [13]. Remarkably, death due to infection was found at a higher frequency after re-K-Tx.

Considering the relevance of infections for transplant-related outcomes, the aim of this study was to describe and compare ID events after f-K-Tx and after re-K-Tx within the same individual. For this, the objective was to describe prospectively collected data on ID events for individuals in the Swiss Transplant Cohort Study (STCS) and to compare ID events after f-K-Tx and re-K-Tx within the same individual, thus reducing the influence of different host factors when performing a matched case-control study. With this, we tested our hypothesis that ID events after f-K-Tx and re-K-Tx differ with respect to the rate of occurrence, pathogen type, and infection site.

## METHODS

### Swiss Transplant Cohort Study

This study was a nested project within the STCS (www.stcs.ch; ClinicalTrials.gov, NCT01204944). The STCS has been prospectively collecting data from all Swiss transplant centers since May 2008 (Basel, Bern, Geneva, St Gallen, Lausanne, and Zurich). Enrollment in the STCS encompasses >93% of all transplant recipients in Switzerland [14]. In predefined time intervals, transplant recipients are followed up by dedicated research assistants to obtain information on the occurrence of ID events.

### Patient Consent Status

Prior to transplantation, written informed consent was obtained for each patient. The STCS was approved by the ethic committees of all participating institutions. In addition, the design of the nested project presented here has been approved by local ethical committees (Kantonale Ethikkommission Zürich, Req 2019-00248).

### Inclusion of ID Events

Uniform predefined criteria for the diagnosis of ID events within the STCS are applied, and the research assistants are supervised by transplant ID physicians. A detailed description of definitions is provided by van Delden et al [15]. For the analysis of ID events in this project, we included the following:

*Proven bacterial infections:* clinically apparent infections combined with detection of the causative bacterium and initiation of targeted antimicrobial treatment; for example, urinary tract infections were defined as the presence of leukocyturia combined with suggestive symptoms, such as fever, urgency, frequency, dysuria, suprapubic tenderness, and the isolation of a causative pathogen in urine cultures with subsequent treatment.
*Symptomatic viral infections:* (1) probable viral disease defined by detection of viral replication combined with symptoms/signs of organ dysfunction, (2) proven viral disease defined by viral detection (either polymerase chain reaction or histopathologic confirmation) in samples gathered from affected organs (eg, biopsy), and (3) viral syndromes defined by viral replication and non–organ-specific clinical signs.
*Proven and probable invasive fungal diseases:* according to criteria from the European Organization for Research and Treatment of Cancer/Invasive Fungal Infections Cooperative Group and the National Institute of Allergy and Infectious Diseases Mycoses Study Group [16].
*Probable infections:* clinical presentations with suspected infectious etiology resulting in the initiation of antimicrobial treatment but with no causative pathogen being identified in routine diagnostics.

Furthermore, infection sites were reported for each ID event and whether the infection required hospitalization. We also collected information on anti-infective prophylactic strategies from all participating transplant centers via a questionnaire.

### Selection of Study Population

**Figure 1.**
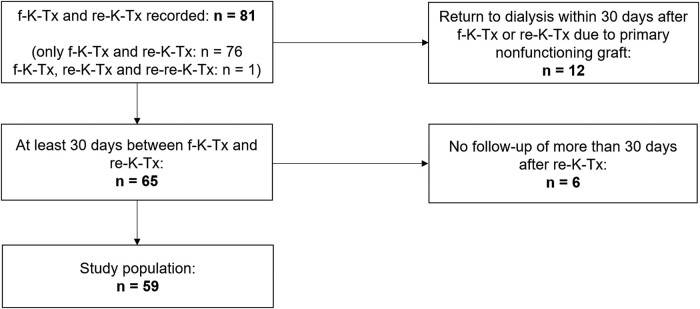
Flowchart of the study population selection. f-K-Tx, first kidney transplantation; re-K-Tx, kidney retransplantation; re-re-K-Tx, third kidney transplantation.

All adult kidney transplant recipients (≥18 years of age at the time of f-K-Tx) who had records on their f-K-Tx and re-K-Tx in the STCS were included in the present study. We excluded patients with re-K-Tx due to a primary nonfunctioning graft, patients with graft loss or return to dialysis within <30 days after transplantation, and patients with <30 days between the serial transplantations. Moreover, we required a follow-up of at least 30 days after re-K-Tx (Figure 1). If patients returned to dialysis after f-K-Tx or re-K-Tx, the follow-up on ID events was paused (until retransplantation), whereas for patients with remaining graft function, the follow-up was continuous.

### Statistical Analysis

#### Descriptive Statistics

The distribution of pathogen types and infection sites of ID events after f-K-Tx and re-K-Tx was compared with a Fisher exact test. The rate of ID events was defined as the number of ID events divided by the sum of person-years of follow-up after f-K-Tx (date of transplantation until graft loss or retransplantation) and re-K-Tx (date of retransplantation until graft loss, death, loss to follow-up, or latest follow-up visit). ID event rates were compared by the rate ratio test (R package *rateratio.test*).

#### Survival Analysis

To account for the different length of follow-up, several survival analysis methods were applied. Time to first ID event after f-K-Tx vs first ID event after re-K-Tx was analyzed through Cox proportional hazards models. The Anderson-Gill counting process was used to compare recurrent ID events in the 2 periods (after f-K-Tx vs after re-K-Tx), once assuming independence of ID events within the same individual and once assuming dependence of ID events within individuals by including the patient identifier as cluster variable. In the multivariable analysis in this case-control setting (with each patient serving as one’s own control before and after re-K-Tx), we adjusted for the most relevant demographic factors based on clinical knowledge and prior literature: time between transplantations (also a measure for increased age after re-K-Tx and calendar year), body mass index [17–19], induction immunosuppression [20, 21], and reason for transplantation being autosomal dominant polycystic kidney disease or other etiology [22]. The timing of ID events was analyzed for all ID events together, as well as for bacterial and viral pathogen types separately.

#### Sensitivity Analysis

We conducted 2 sensitivity analyses: (1) restricting to ID events that required hospitalization and (2) excluding urinary tract infections.

Data analysis and statistical testing were performed with R Statistical Software (R version 4.2.1).

## RESULTS

### Study Population

In total, 59 patients were included, with a median age of 47 years (range, 18–73) at f-K-Tx and with 59.3% being male (Table 1). The most common underlying causes for end-stage renal disease were glomerulonephritis (n = 22, 37.3%) and autosomal dominant polycystic kidney disease (n = 13, 22.0%). Prior to f-K-Tx, 46 (78.0%) patients received renal replacement therapy with intermittent hemodialysis and 16 (27.1%) with peritoneal dialysis. Most patients (n = 37, 62.7%) received grafts from deceased donors. The median period between f-K-Tx and re-K-Tx was 5.1 years (range, 0.4–12.8).

**Table 1.** Study Population (N = 59)

Baseline Characteristic	No. (%) or Median (Range)	
Gender		
Male	35 (59.3)	
Female	24 (40.7)	
Ethnicity		
Caucasian	53 (89.8)	
Asian	3 (5.1)	
African	1 (1.7)	
Other or unknown	2 (3.4)	
Underlying cause of end-stage renal disease		
Glomerulonephritis	22 (37.3)	
ADPKD	13 (22.0)	
Nephrosclerosis	7 (11.9)	
Reflux	4 (6.8)	
Other/unknown	13 (22.0)	
Years between		
First and retransplantation	5.1 (0.4–12.8)	
Graft loss and retransplantation	1.7 (0.1–5.9)	

Transplant-Related Characteristic	First Transplantation	Retransplantation
At transplantation		
Age	47 (18–73)	53 (18–73)
Body mass index	25.6 (17.6–36.7)	24.8 (16.6–36.7)
Years of follow-up time after transplantation	2.5 (0.1–11.2)	2.0 (0.1–10.0)
Renal replacement therapy before transplantation		
Hemodialysis	46 (78.0)	47 (79.7)
Peritoneal dialysis	16 (27.1)	8 (14.5)
None	14 (23.7)	14 (23.7)
Years of renal replacement therapy before transplantation	2.5 (0.1–15.0)	1.7 (0.1–6.7)
Donation type		
Donation after brain death	37 (62.7)	45 (76.3)
Living related	12 (20.3)	3 (5.1)
Living unrelated	6 (10.2)	7 (11.9)
Donation after circulatory death	4 (6.8)	2 (3.4)
AB0-incompatible donor	2 (3.4)	1 (1.7)
Induction immunosuppression		
Basiliximab	46 (66.7)	39 (61.9)
Thymoglobulin/ATG	20 (29.0)	18 (28.6)
Maintenance immunosuppression^a^		
Tacrolimus-containing regimen	37 (62.7)	51 (86.4)
Cyclosporine A–containing regimen	19 (32.2)	12 (20.3)
MMF or EC-MPS	53 (89.9)	44 (74.6)
Glucocorticoid	54 (91.5)	49 (83.1)
Other/unknown	7 (11.8)	11 (18.6)
Comorbidities		
Diabetes	6 (10.2)	15 (25.4)
Hypertension	46 (78.0)	47 (79.7)
Dyslipidemia	23 (39.0)	29 (49.2)

Abbreviations: ADPKD, autosomal dominant polycystic kidney disease; ATG, antithymocyte globulin; EC-MPS, enteric-coated mycophenolate sodium; MMF, mycophenolate mofetil.

^a^Maintenance immunosuppressive regimen started within the first 2 weeks after first kidney transplantation and after kidney retransplantation.

### Prophylactic Strategies

Regarding anti-infective prophylactic strategies, 5 transplant centers prescribed trimethoprim/sulfamethoxazole (80/400 mg) every day for 6 months and 1 center for 12 months; no center used routine antifungal prophylaxis. For cytomegalovirus prevention,

- In high-risk constellation (donor+, recipient–), all centers used a prophylactic strategy with administration of valganciclovir.
- In intermediate-risk constellation (donor+, recipient+; donor–, recipient+), 2 of 6 centers used an universal prophylactic strategy with administration of valganciclovir, and 4 of 6 centers used a preemptive approach—except if antithymocyte globulin was used for induction, in which case a prophylactic strategy with administration of valganciclovir was applied.
- In low-risk constellation (donor–, recipient–), 5 of 6 centers used a preemptive approach and 1 of 6 centers a prophylactic approach with administration of valacyclovir.

If a preemptive approach for cytomegalovirus was chosen, 5 of 6 centers did not administer prophylaxis for herpes simplex virus (HSV) or varicella zoster virus (VZV); 1 of 6 centers prescribed valacyclovir for HSV/VZV prophylaxis in AB0-incompatible kidney transplantation and a high risk constellation for HSV (donor +, recipient–). Routine perioperative antibiotic prophylaxis consisted of administration of a single dose of amoxicillin/clavulanate (1 center), cefuroxime (3 centers), ceftriaxone (1 center), or piperacillin/tazobactam (1 center) within 30 to 60 minutes before incision [23]. All centers reported identical prophylactic strategies for f-K-Tx and re-K-Tx. Posttransplant BK virus surveillance differed among transplant centers; however, most centers screened for BK virus in plasma samples (Supplementary Table 1).

### Number and Timing of ID Events

**Figure 2.**
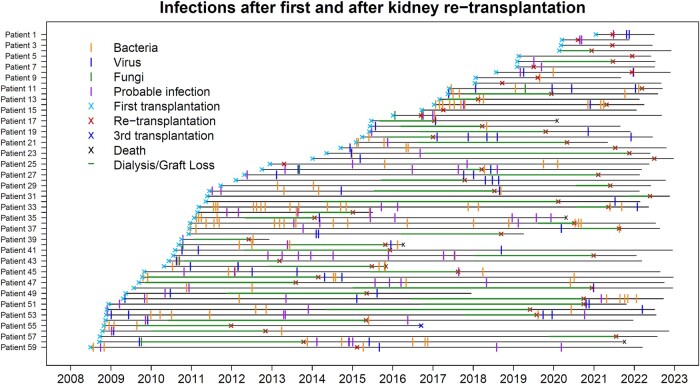
Timeline of all patients included in the study population: each horizontal line corresponds to 1 patient. Observation time starts with first kidney transplantation and ends with the latest follow-up information, or a third transplantation or death (cross at the end of the horizontal line). All infectious disease events (vertical lines) are indicated: bacterial, viral, fungal, and probable infections without identification of causative pathogens. See Supplementary Figure 1 for restriction to infections requiring hospitalization. The time points of the transplantations are indicated by crosses.

In total, 312 ID events in 52 patients were observed; 7 patients did not have an ID event after f-K-Tx and neither after re-K-Tx. For 129 ID events in 40 patients, hospitalization was required. Among patients with at least 1 ID event, 42 (71.2%) had ID events after f-K-Tx and 39 (66.1%) after re-K-Tx. See Figure 2 for all ID events of the whole study population and Supplementary Figure 2 for the timeline of ID events requiring hospitalization. An overall 193 ID events occurred after f-K-Tx (0.95/person-year of follow-up) and 119 after re-K-Tx (0.67/person-year of follow-up, *P* = .003). For ID events requiring hospitalization, the rate was still lower after re-K-Tx (54 ID events, 0.30/person-year of follow-up) than after f-K-Tx (75 ID events, 0.37/person-year of follow-up), although this difference was not statistically significant (*P* = .32). In the multivariable “time to first event” analysis, no difference was observed between ID events after f-K-Tx and re-K-Tx or for the pathogen types separately. When recurrent events were modeled, the rate was significantly lower for ID events after re-K-Tx, whether assuming independence of ID events (hazard ratio, 0.70; *P* = .02) or assuming dependence (hazard ratio, 0.70; *P* = .009). A reduced hazard of ID events was seen for bacterial infections but not for viral infections (Supplementary Figure 3). For ID events requiring hospitalization, the trend (ie, lower hazard for ID events after re-K-Tx) was similar but not significant (Supplementary Figure 4). Urinary tract infections were the most frequent infection site, particularly after f-K-Tx; hence, we performed a sensitivity analysis excluding urinary tract infections. Again, a lower hazard was observed for ID events after re-K-Tx (Supplementary Figure 5).

### Causative Pathogens in ID Events

**Figure 3.**
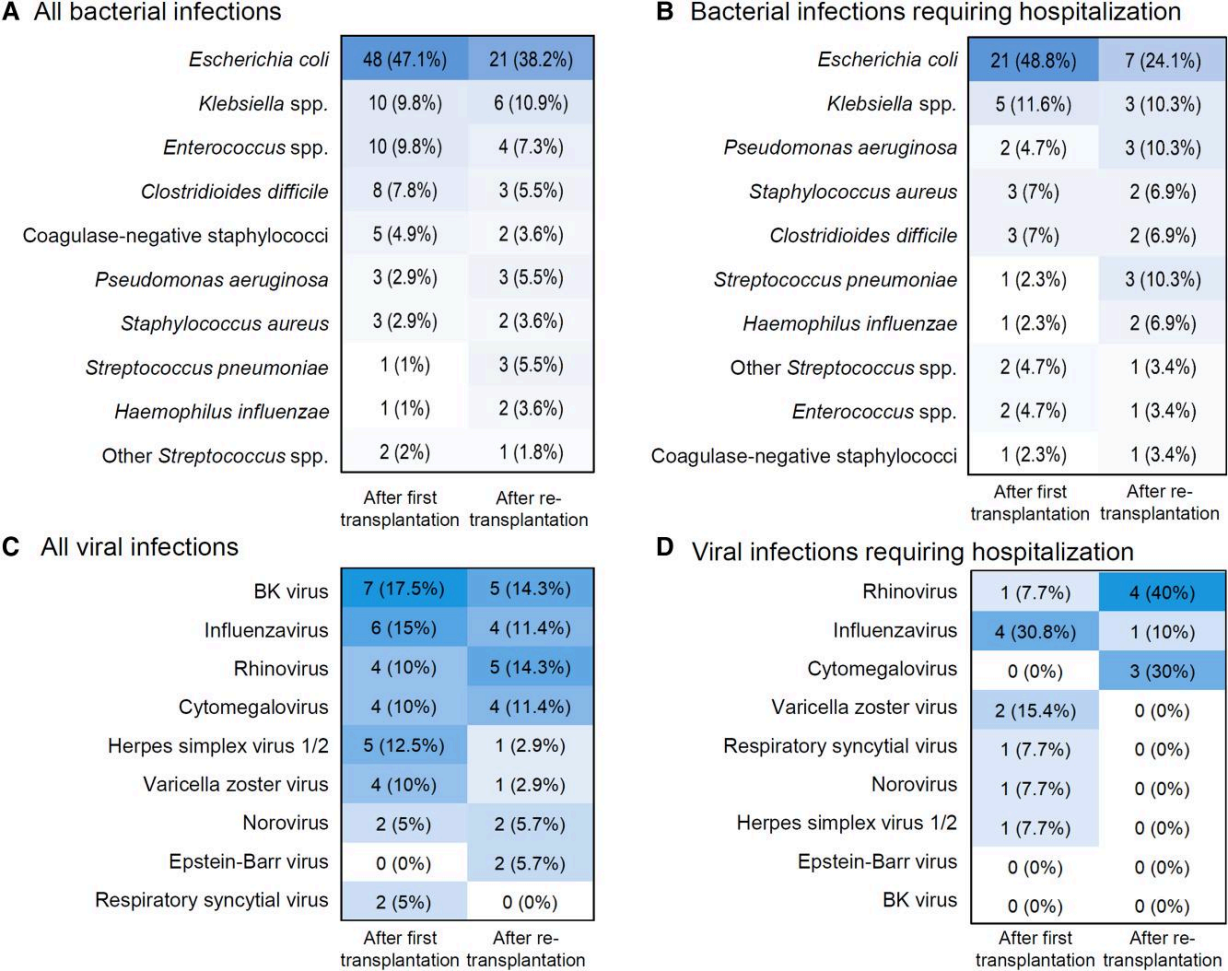
Causative bacteria and viruses for all study patients (*A* and *C*), as well as restriction to infectious disease events requiring hospitalization (*B* and *D*). Figures display the absolute number (percentage) of the 10 most commonly detected bacteria and viruses.

Among infections with detection of a causative pathogen, bacterial infections were most common (n = 157, 65.7%), followed by viral (n = 75, 31.4%) and fungal (n = 7, 2.9%) infections. There were 73 (23.4%) probable ID events—specifically, clinical scenarios with suspected infectious etiology prompting empiric anti-infective treatment—but routine diagnostics did not identify a causative pathogen. Among bacteria, *Escherichia coli* (n = 69, 43.9%), *Klebsiella* spp (n = 16, 10.2%), and *Enterococcus* spp (n = 14, 8.9%) predominated. Among viral infections, BK virus (n = 12, 16.0%), influenza virus (n = 10, 13.3%), and rhinovirus (n = 9, 12.0%) were most common. See Figure 3 for all pathogen types (all ID events as well as those restricted to requiring hospitalization). Among ID events with detection of a causative pathogen, a significantly different distribution between f-K-Tx and re-K-Tx was found: we observed more bacterial infections (68.9% vs 60.4%) and fungal infections (4.0% vs 1.1%, *P* = .11) after f-K-Tx but fewer viral infections (27.0% vs 38.5%). Regarding bacteria, *E coli*, *Enterococcus* spp, *Clostridioides difficile*, and coagulase-negative staphylococci were more frequent after f-K-Tx, whereas *Pseudomonas aeruginosa* was more often found after re-K-Tx. Among viral infections, BK virus, influenza virus, HSV, and VZV were more common after f-K-Tx. A single fungal infection caused by *Candida* non-*albicans* (probable infection, urinary tract) was observed after re-K-Tx, whereas the following fungal infections were reported after f-K-Tx: 2 *Aspergillus* spp (proven disease, both respiratory tract), 1 *Candida albicans* (probable disease, mucocutaneous), 1 *C* non-*albicans* (proven disease, gastrointestinal), 1 *Pneumocystis jirovecii* infection (probable disease, respiratory tract), and 1 *Alternaria* spp (proven disease, respiratory tract).

### Infection Sites

**Figure 4.**
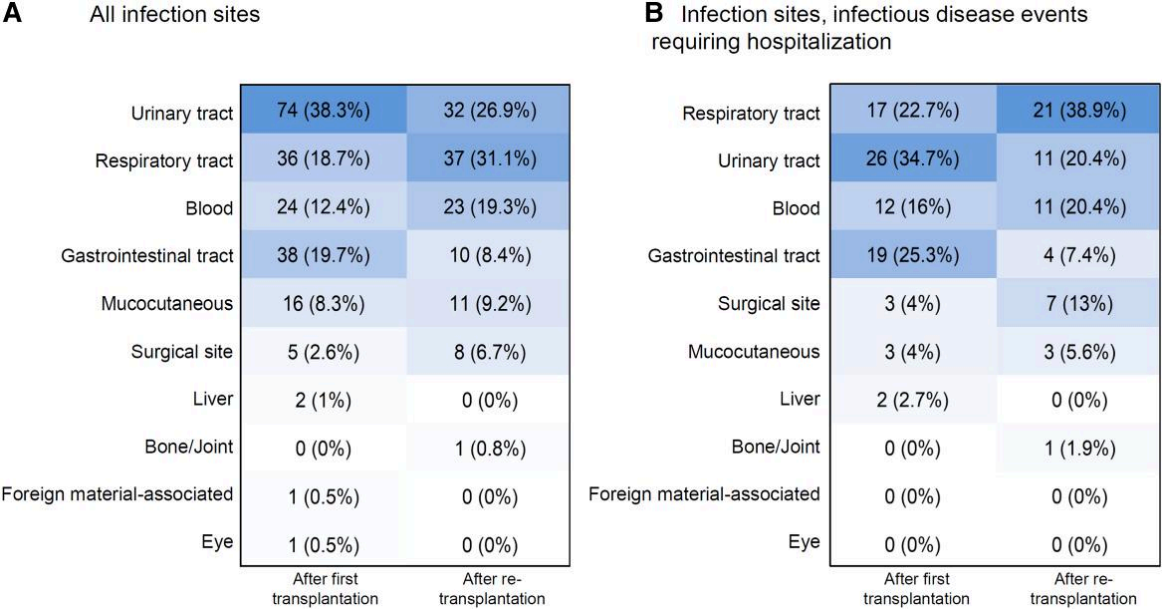
Infection sites for all infectious disease events (*A*) and restriction to infectious disease events requiring hospitalization (*B*). Figures display the absolute number (percentage) of the 10 most common infection sites.

The most frequent infection sites were the urinary tract (n = 106, 34.0%), followed by respiratory tract (n = 73, 23.4%), gastrointestinal (n = 48, 15.4%), and bloodstream (n = 47, 15.1%). In 33 ID events, the infection affected >1 site (eg, 10 urinary tract infections presented with bacteremia). In a comparison of the infection sites, we detected significant differences between f-K-Tx and re-K-Tx (Figure 4). After f-K-Tx the urinary and gastrointestinal tracts were more often affected, whereas after re-K-Tx the respiratory tract and surgical site were more often affected (*P* < .001).

## DISCUSSION

In the present study encompassing 59 kidney transplant recipients who received a re-K-Tx, we observed a higher infection rate after f-K-Tx as compared with re-K-Tx. Causative pathogens and site of infections differed between f-K-Tx and re-K-Tx.

Our result of more frequent ID events after f-K-Tx vs the period after re-K-Tx might appear counterintuitive at a first glance. Patients with re-K-Tx have been receiving immunosuppressive therapy since f-K-Tx. The reasons for the lower infection rate after re-K-Tx remain speculative. It can be hypothesized that improved organ function after retransplantation might have some protective effect against infections. Another hypothesis can be that individuals receiving a re-K-Tx needed to prove compliance after f-K-Tx to be considered for retransplantation. Compliance with anti-infective prophylaxis is crucial for prevention of ID events after any transplantation.

Most ID events were caused by bacteria after f-K-Tx and re-K-Tx. *Enterobacterales*, followed by *Enterococcus* spp, contributed to the majority of bacterial infections. Similarly, van Delden et al found *Enterobacterales* and *Enterococcus* spp as most common bacterial pathogens in the first year after kidney transplantation [15]. In our patient population, most viral infections were caused by BK virus, influenza, rhinovirus, and herpesviruses. Van Delden et al reported HSV, followed by cytomegalovirus, BK virus, VZV, and influenza, as the most frequent viral infections in the first year after kidney transplantation. VZV infections contributed with approximately 6.7% to viral infections in our study and were more common after f-K-Tx as compared with re-K-Tx.

More fungal infections were observed after f-K-Tx than after re-K-Tx. A single *Candida* spp infection was observed after re-K-Tx, whereas 2 *Candida* spp infections, 3 mold infections, and 1 *P jirovecii* occurred after f-K-Tx. In our prior study on liver retransplantation, fungal infections caused by *Candida* spp decreased, whereas fungal infections caused by *Aspergillus* spp increased after retransplantation [24]. Several studies reported urinary tract infections as the most common infection in kidney transplant recipients [25–27]. Interestingly, we observed more urinary tract infections after f-K-Tx than re-K-Tx. The reason for this finding remains unclear. There were more surgical site infections after re-K-Tx. It can be hypothesized that surgical site infections are more likely if an access route via a site of prior surgery is chosen. However, this information was not routinely collected, hindering further analysis of this hypothesis.

After re-K-Tx, respiratory tract infections were more often reported. This finding resembles a prior study analyzing ID events in liver retransplantation; the authors also described a higher frequency of respiratory tract infection after liver retransplantation [24].

Strengths of this study include the multicentric design and the exclusive use of prospectively collected data. Although the total population size in this study is small, it needs to be acknowledged that the data collection of the STCS is highly representative for transplantations performed in Switzerland, including 93% of all transplantations performed there since 2008 [14].

Our study has some limitations that must be acknowledged. If patients returned to dialysis, the data collection on ID events was discontinued. Thus, the period of graft failure after first transplantation until retransplantation was censored. However, if a patient returns to dialysis, immunosuppression will be usually tapered. Our study likely reflects the clinically most relevant periods of intense immunosuppression. We also aimed to overcome this limitation by reporting rates, thereby reflecting observation times for ID events, and by adjusting for these periods in time-to-event analysis (ie, after f-K-Tx, dialysis, after re-K-Tx). Our data set included only center-specific prophylactic strategies, not data at the individual level. Thus, a more detailed analysis of the prophylactic strategies and their associations with ID events could not be performed. Similarly, we do not have information on the individual compliance with prophylactic treatments and vaccinations administered. It can be hypothesized that patients listed for a re-K-Tx represent a selected subset of transplant recipients who had to prove compliance with previously prescribed drugs. A significant lower hazard for infections after re-K-Tx was found in the pooled analysis of all ID events. For this analysis, all ID events independent of the causative pathogen were treated equally, even if the clinical relevance might have been different. To address this limitation, we performed several sensitivity analyses that, with exception of the subset of viral infections, supported a trend of lower hazards after re-K-Tx. There might be some underreporting in self-limiting ID events, such as viral respiratory tract or gastrointestinal infections. However, whether there is a difference in reporting of self-limiting diseases after re-K-Tx and f-K-Tx remains hypothetic.

## CONCLUSIONS

Infections were less frequent after re-K-Tx as compared with f-K-Tx. Significant differences of infection sites were observed between f-K-Tx and re-K-Tx. These findings might influence future prophylactic treatment strategies following retransplantation.

## Supplementary Material

ofae055_Supplementary_Data

## Supplementary Data

Supplementary materials are available at *Open Forum Infectious Diseases* online. Consisting of data provided by the authors to benefit the reader, the posted materials are not copyedited and are the sole responsibility of the authors, so questions or comments should be addressed to the corresponding author.

## Notes

***Acknowledgments.*** We thank all patients for their willingness to participate in the STCS. We thank the reviewers for valuable input leading to substantial improvement of this project.

***Members of the STCS.*** P. Amico, J.-D. Aubert, V. Banz, S. Beckmann, G. Beldi, C. Berger, E. Berishvili, A. Berzigotti, I. Binet, P.-Y. Bochud, S. Branca, H. Bucher, E. Catana, A. Cairoli, Y. Chalandon, S. De Geest, O. De Rougemont, S. De Seigneux, M. Dickenmann, J. L. Dreifuss, M. Duchosal, T. Fehr, S. Ferrari-Lacraz, C. Garzoni, D. Golshayan, N. Goossens, F. H. J. Halter, D. Heim, C. Hess, S. Hillinger, H. H. Hirsch, P. Hirt, G. Hofbauer, U. Huynh-Do, F. Immer, M. Koller, M. Laager, B. Laesser, F. Lamoth, R. Lehmann, A. Leichtle, O. Manuel, H. P. Marti, M. Martinelli, V. McLin, K. Mellac, A. Merçay, K. Mettler, A. Müller, N. J. Mueller, U. Müller-Arndt, B. Müllhaupt, M. Nägeli, G. Oldani, M. Pascual, J. Passweg, R. Pazeller, K. Posfay-Barbe, J. Rick, A. Rosselet, S. Rossi, S. Rothlin, F. Ruschitzka, T. Schachtner, U. Schanz, S. Schaub, A. Scherrer, A. Schnyder, M. Schuurmans, S. Schwab, T. Sengstag, F. Simonetta, S. Stampf, J. Steiger, G. Stirnimann, U. Stürzinger, C. Van Delden, J.-P. Venetz, J. Villard, J. Vionnet, M. Wick, M. Wilhelm, P. Yerly.

***Financial support.*** This work was supported by the academic career program Filling the Gap of the Medical Faculty of the University of Zurich (to P. W. S.). This study was performed in the framework of the Swiss Transplant Cohort Study, supported by the Swiss National Science Foundation, the Swiss University Hospitals (G15), and transplant centers.
