# Supplementary material for: Do Infectious Diseases After Kidney Retransplantation Differ From Those After First Kidney Transplantation?
Source: Open Forum Infect Dis. 2024 Feb 6;11(3):ofae055. doi: 10.1093/ofid/ofae055 (PMC10923290; doi:10.1093/ofid/ofae055)

**Supplementary Material**

**Do infectious diseases after kidney re-transplantation differ from those after first kidney transplantation?**

Katharina Kusejko^1,2^, Dionysios Neofytos^3^, Christian van Delden^3^, Hans H. Hirsch^4,5^, Pascal Meylan^6^, Katia Boggian^7^, Cedric Hirzel^8^, Christian Garzoni^8,9^, Daniel Sidler^10^, Aurelia Schnyder^11^, Stefan Schaub^12^, Déla Golshayan^13^, Fadi Haidar^14^, Marco Bonani^15^, Roger D. Kouyos^1,2^, Nicolas J. Mueller^1^, Peter W. Schreiber^1^ and the Swiss Transplant Cohort Study

**Affiliations**

^1^ Department of Infectious Diseases and Hospital Epidemiology, University Hospital Zurich, Zurich

^2^ Institute of Medical Virology, University of Zurich, Zurich, Switzerland

^3^ Division of Infectious Diseases, University Hospital of Geneva, Geneva, Switzerland

^4^ Transplantation & Clinical Virology, Department Biomedicine, University of Basel, Basel, Switzerland

^5^ Clinical Virology, Laboratory Medicine / Infectious Diseases & Hospital Epidemiology, University Hospital Basel, Basel, Switzerland

^6^ Faculty of Biology and Medicine, University of Lausanne, Lausanne, Switzerland

^7^ Division of Infectious Diseases, Infection Prevention and Travel Medicine, Cantonal Hospital of St. Gallen, St. Gallen, Switzerland

^8^ Department of Infectious Diseases, Inselspital, Bern University Hospital, University of Bern, Bern, Switzerland

^9^ Department of Internal Medicine, Clinica Luganese Moncucco, Lugano, Switzerland

^10^ Division of Nephrology and Hypertension, Inselspital, Bern University Hospital, Bern, Switzerland

^11^ Clinic for Nephrology, Cantonal Hospital of St. Gallen, St.Gallen, Switzerland

^12^ Clinic for Transplantation Immunology and Nephrology, University Hospital Basel, Basel, Switzerland.

^13^ Transplantation Center, Lausanne University Hospital, Lausanne, Switzerland

^14^ Division of Nephrology, Department of Medicine, University Hospital of Geneva, Geneva, Switzerland

^15^ Division of Nephrology, University Hospital Zurich, Zurich, Switzerland

**Supplementary table 1:** Posttransplant BK virus surveillance strategies

| Center | Center 1 | Center 2 | Center 3 | Center 4 | Center 5 | Center 6 |
| --- | --- | --- | --- | --- | --- | --- |
| Screening strategy | Plasma BK virus PCR at month 1, 2, 3, 4, 5, 6, 8, 10, 12, 18 and 24, then annually. | Urine screening for Decoy cells every 2 weeks in the first 3 months, afterwards in month 4, 5, 6 and 12, then annually.  → if ≥ 3 Decoy cells/10 HPF, Plasma BK virus PCR | Plasma BK Virus PCR at month 1, 2, 3, 4, 5 and 6, then annually | Plasma BK Virus PCR monthly in the first year, every 3 months in the second year, then annually | Plasma BK Virus PCR at month 2, 4, 6, 9 and 12, then annually | Plasma BK Virus PCR weekly in the first year |

**Supplementary Figure 1: Timeline of all patients included in the study population restricted to ID events requiring hospitalization:** Each horizontal line corresponds to one patient. Observation time starts with first kidney transplantation and ends with the latest follow‐up information. All infectious disease events are indicated: bacterial (orange), viral (blue), fungal (green), and probable infections without identification of causative pathogens (purple). The time points of the transplantations are indicated by crosses (light blue, first kidney transplantation; red, kidney re-transplantation; dark blue, third kidney transplantation). Death is indicated with a black cross.


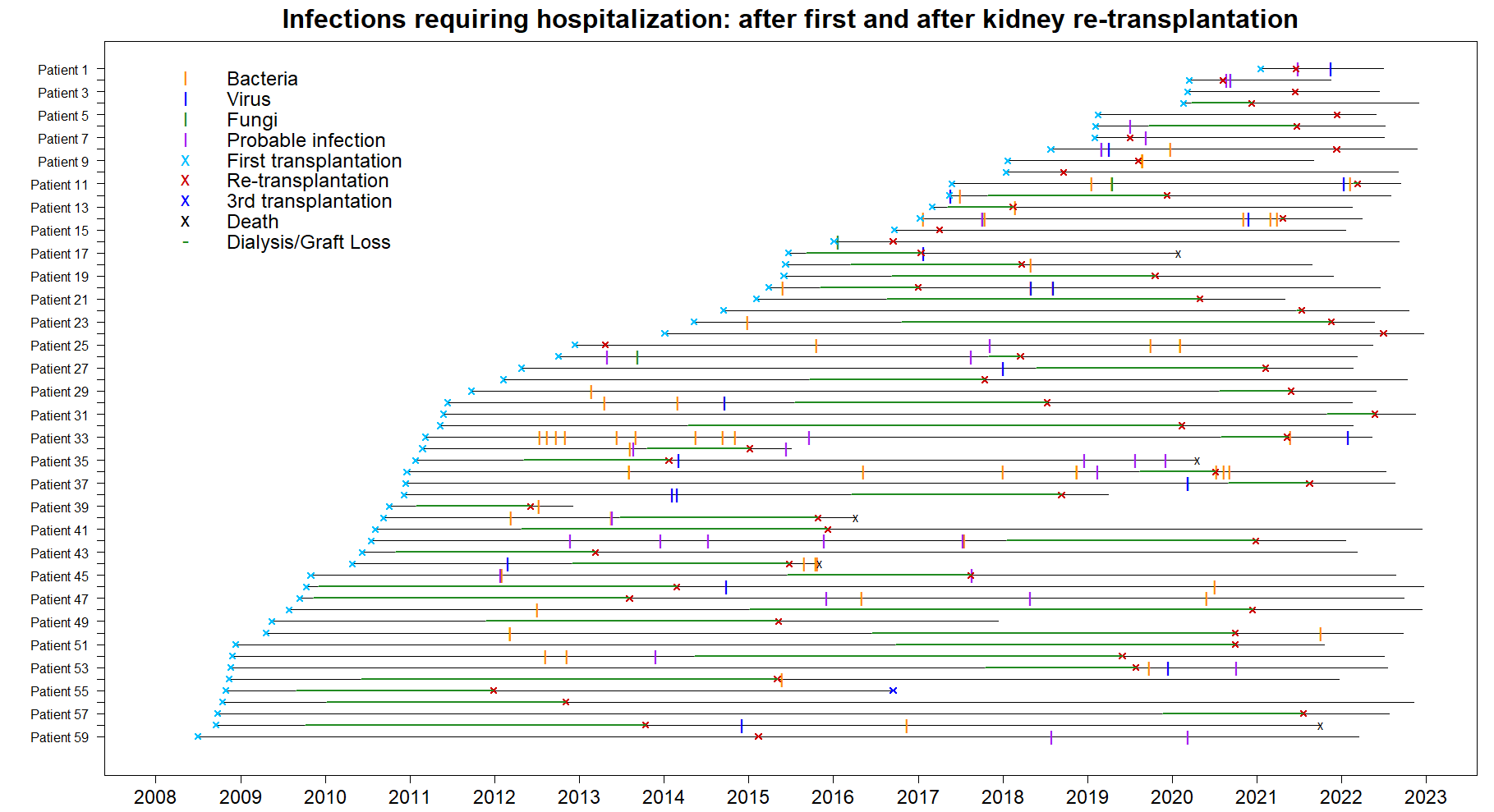
**Supplementary Figure 2**: Timing of ID events.


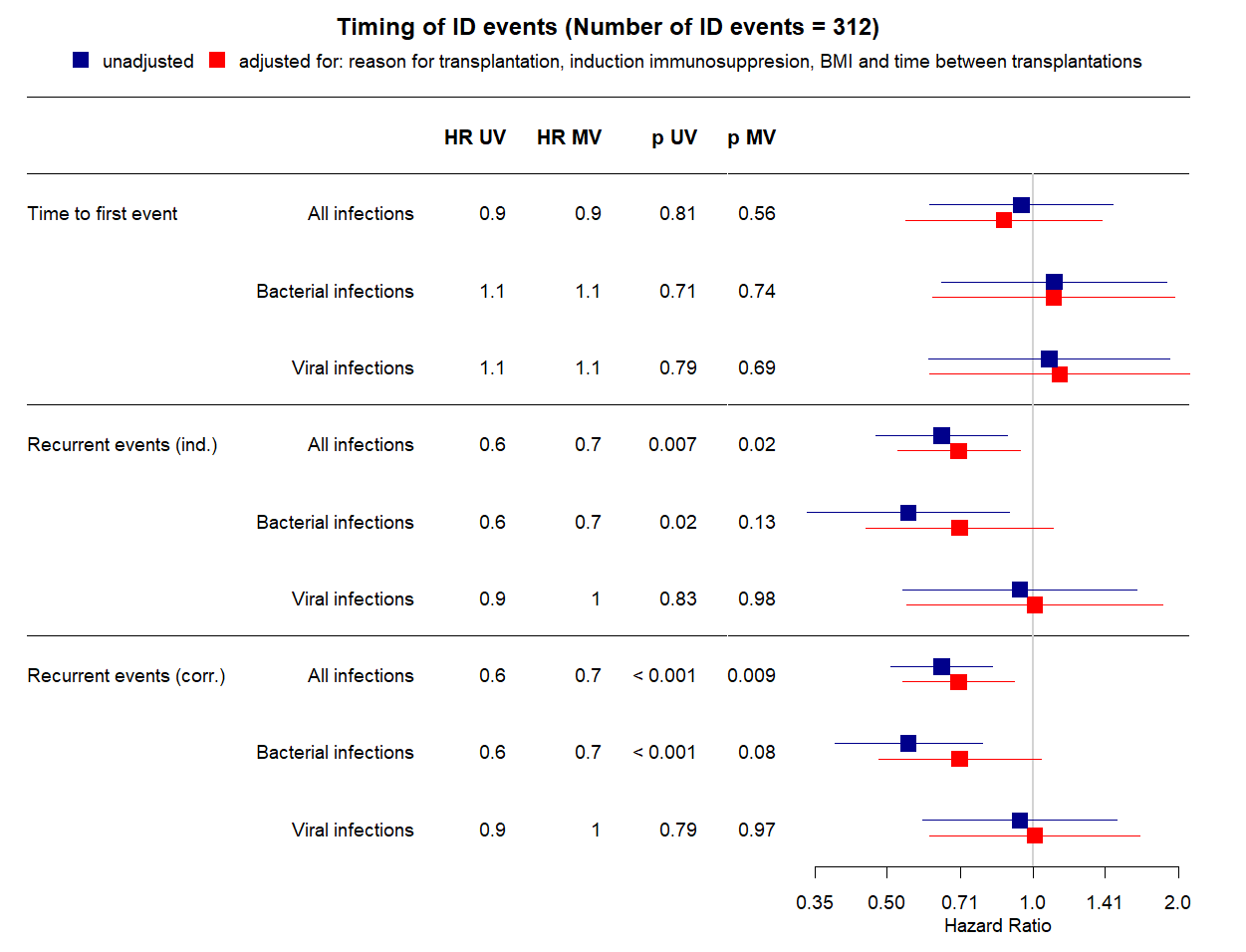


**Supplementary Figure 3:** Timing of ID events, restricted to ID events requiring hospitalization.


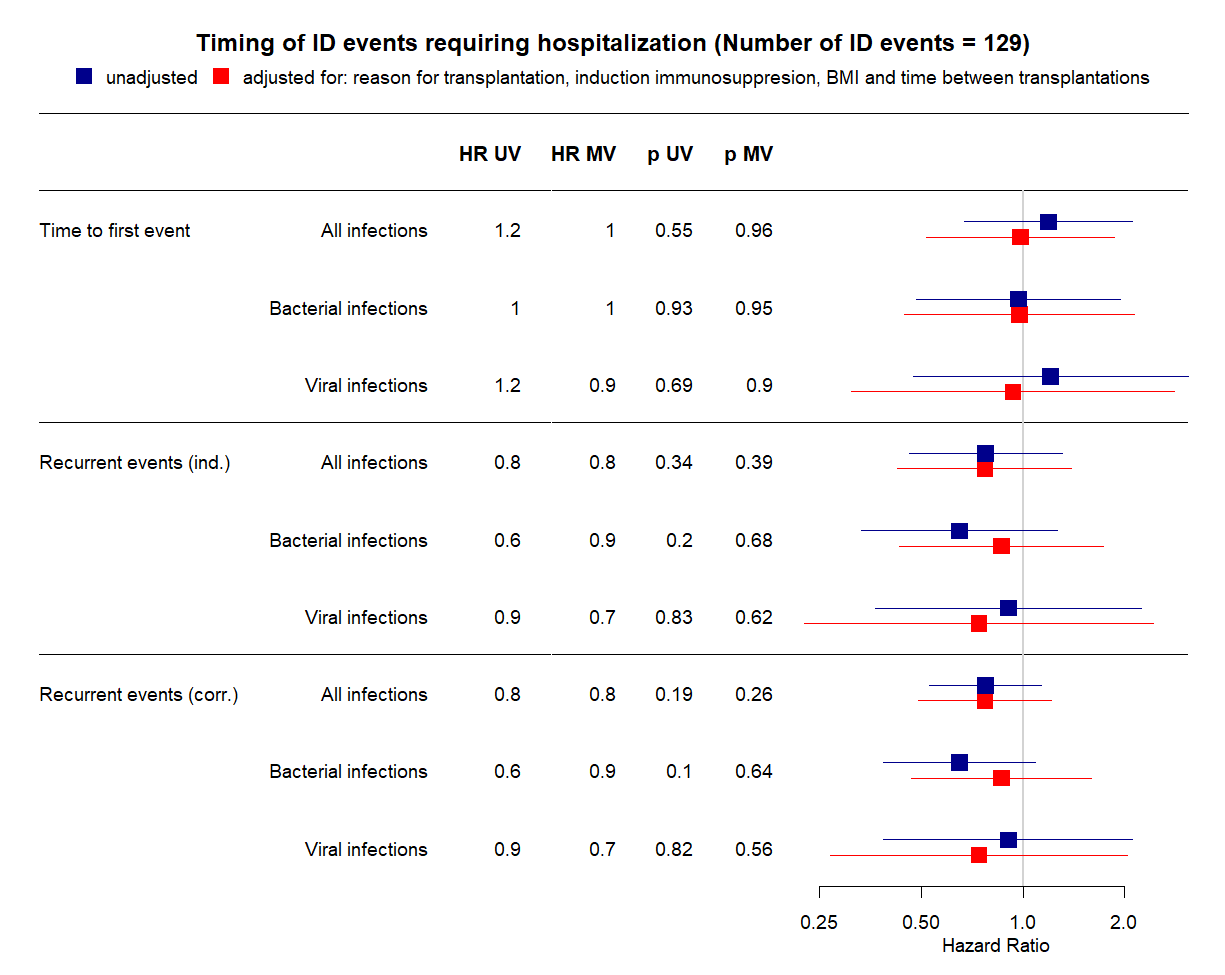


**Supplementary Figure 4:** Timing of ID events, excluding urinary tract infections.


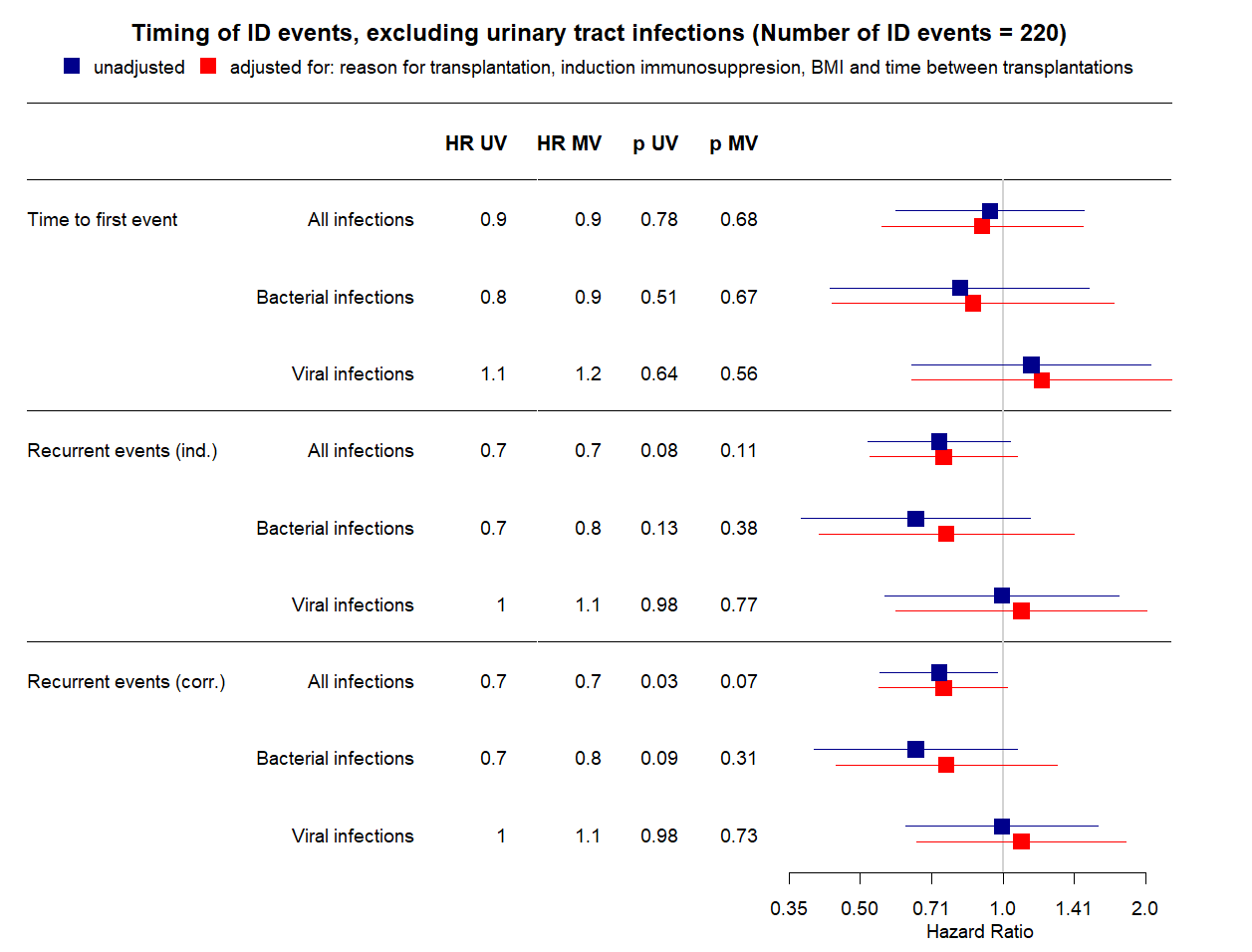

Supplement: ofae055_Supplementary_Data [file ofae055_supplementary_data.docx]
